# Bis(2-amino-3-carb­oxy­pyrazin-1-ium) sulfate dihydrate

**DOI:** 10.1107/S1600536811005824

**Published:** 2011-02-23

**Authors:** Fadila Berrah, Amira Ouakkaf, Sofiane Bouacida, Thierry Roisnel

**Affiliations:** a Laboratoire de Chimie Appliquée et Technologie des Matériaux LCATM, Université Larbi Ben M’Hidi, 04000 Oum El Bouaghi, Algeria; b Département Sciences de la Matière, Faculté des Sciences Exactes et Sciences de la Nature et de la Vie, Université Larbi Ben M’hidi, 04000 Oum El Bouaghi, Algeria; c Unité de Recherche de Chimie de l’Environnement et Moléculaire Structurale, CHEMS, Faculté des Sciences Exactes, Université Mentouri Constantine 25000, Algeria; d Centre de Difractométrie X, UMR 6226 CNRS Unité Sciences Chimiques de Rennes, Université de Rennes I, 263 Avenue du Général Leclerc, 35042 Rennes, France

## Abstract

The crystal structure of the title compound, 2C_5_H_6_N_3_O_2_^+^·SO_4_^2−^·2H_2_O, displays a variety of N—H⋯O and O—H⋯O hydrogen bonds in which all potential donors and acceptors are involved. In the crystal, cations and anions are inter­connected, forming *R*_3_^3^(10) and *R*_2_^2^(8) ring motifs whereas the anions and water mol­ecules form *R*_2_^3^(10) rings, which develop in chains running along [100]. The resulting three-dimensional network exhibits undulating sheets parallel to (011), marked by the presence of *R*_6_^6^(26) rings in which six cations are involved.

## Related literature

For related compounds, see: Berrah *et al.* (2005*a* ▶,*b* ▶, 2011 ▶); Bouacida *et al.* (2005 ▶, 2009 ▶); Dobson & Gerkin (1996 ▶). For hydrogen-bond motifs, see: Bernstein *et al.* (1995 ▶); Etter *et al.* (1990 ▶). For similar inter­molecular inter­actions, see: Dorn *et al.* (2005 ▶), Janiak (2000 ▶); Desiraju (2003 ▶).
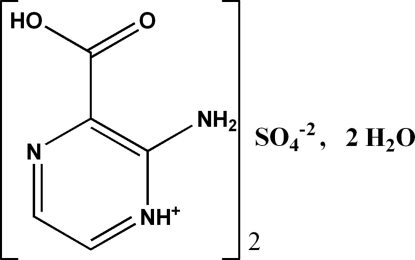


## Experimental

### 

#### Crystal data

2C_5_H_6_N_3_O_2_^+^·SO_4_^2−^·2H_2_O
*M* *_r_* = 412.36
Monoclinic, 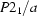

*a* = 7.7214 (4) Å
*b* = 20.7043 (14) Å
*c* = 10.6398 (7) Å
β = 109.299 (2)°
*V* = 1605.36 (17) Å^3^
*Z* = 4
Mo *K*α radiation
μ = 0.27 mm^−1^
*T* = 150 K
0.55 × 0.36 × 0.15 mm

#### Data collection

Bruker APEXII diffractometer
Absorption correction: multi-scan (*SADABS*; Sheldrick, 2002 ▶) *T*_min_ = 0.708, *T*_max_ = 0.960
13466 measured reflections
3675 independent reflections
3146 reflections with *I* > 2σ(*I*)
*R*_int_ = 0.039

#### Refinement

*R*[*F* ^2^ > 2σ(*F* ^2^)] = 0.035
*wR*(*F* ^2^) = 0.096
*S* = 1.03
3675 reflections
246 parameters
H-atom parameters constrained
Δρ_max_ = 0.47 e Å^−3^
Δρ_min_ = −0.48 e Å^−3^

### 

Data collection: *APEX2* (Bruker, 2001 ▶); cell refinement: *SAINT* (Bruker, 2001 ▶); data reduction: *SAINT*; program(s) used to solve structure: *SIR2002* (Burla *et al.*, 2003 ▶); program(s) used to refine structure: *SHELXL97* (Sheldrick, 2008 ▶); molecular graphics: *ORTEP-3 for Windows* (Farrugia, 1997 ▶) and *DIAMOND* (Brandenburg & Berndt, 2001 ▶); software used to prepare material for publication: *WinGX* (Farrugia, 1999 ▶).

## Supplementary Material

Crystal structure: contains datablocks global, I. DOI: 10.1107/S1600536811005824/dn2656sup1.cif

Structure factors: contains datablocks I. DOI: 10.1107/S1600536811005824/dn2656Isup2.hkl

Additional supplementary materials:  crystallographic information; 3D view; checkCIF report

## Figures and Tables

**Table 1:** Hydrogen-bond geometry (Å, °)

*D*—H⋯*A*	*D*—H	H⋯*A*	*D*⋯*A*	*D*—H⋯*A*
N1*A*—H1*A*1⋯O4	0.88	1.92	2.7970 (18)	175
N1*A*—H1*A*2⋯O6*A*	0.88	2.04	2.6741 (18)	128
N1*A*—H1*A*2⋯O6*B*^i^	0.88	2.30	3.0158 (18)	138
N2*A*—H2*A*⋯O1	0.88	1.83	2.6915 (18)	167
N1*B*—H1*B*1⋯O2	0.88	2.34	3.0827 (18)	142
N1*B*—H1*B*2⋯O6*B*	0.88	2.08	2.7144 (19)	129
N1*B*—H1*B*2⋯O6*A*^ii^	0.88	2.10	2.8237 (18)	139
N2*B*—H2*B*⋯O1*W*	0.88	1.81	2.6705 (18)	167
O5*B*—H5*B*⋯O2*W*	0.84	1.67	2.5046 (17)	174
O5*A*—H5*A*⋯O2^i^	0.84	1.78	2.6192 (16)	175
O1*W*—H1*W*⋯O3	0.85	1.95	2.7934 (19)	175
O1*W*—H2*W*⋯O2^iii^	0.85	2.06	2.8996 (18)	167
O1*W*—H2*W*⋯O4^iii^	0.85	2.65	3.2856 (17)	133
O2*W*—H3*W*⋯O4^ii^	0.85	1.88	2.7351 (17)	177
O2*W*—H4*W*⋯O3^iv^	0.86	1.91	2.7633 (17)	171

Symmetry codes: (i) 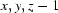; (ii) 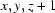; (iii) 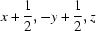; (iv) 
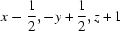
.

**Fig. 1.**
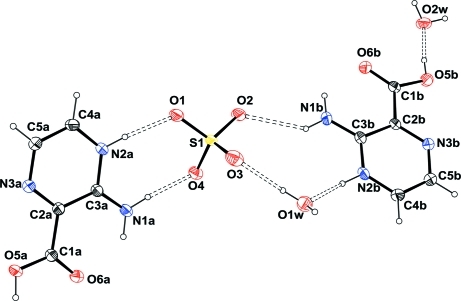
The asymmetric unit of the title compound with the atomic labelling scheme.Displacement are drawn at the 50% probability level. H atoms are represented as small spheres of arbitrary radii. Hydrogen bonds are shown as dashed lines.

**Fig. 2.**
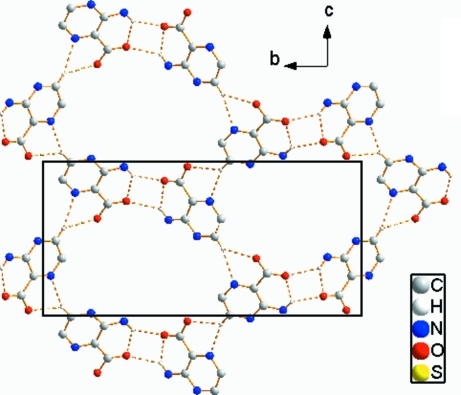
Partial packing view showing undulating sheets parallel to (011) plane and R66(26) rings set motif. Hydrogen bonds are shown as dashed lines. Hydrogen atoms not involved in H-bonds have been omitted for clarity

**Fig. 3.**
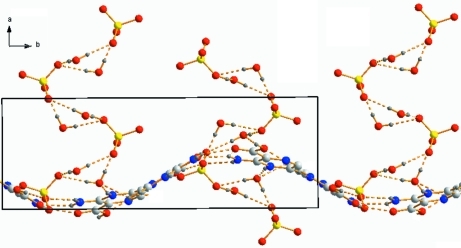
Partial packing view showing sulfate-water chains extending along [100] direction and undulating sheets. Hydrogen bonds are shown as dashed lines.Hydrogen atoms not involved in H-bonds have been omitted for clarity.

## supplementary crystallographic information

### Comment

Hydrogen bonds are object of several studies, that aim at elucidate their influence on crystal construction and compounds properties (Desiraju, 2003). N-heterocyclic compounds such as pyrazine and its derivatives may be interesting units to built new edifices involving original hydrogen-bonding scheme since they include a variety of potential hydrogen donors and acceptors. In this perspective and as a part of our search for new hybrid compounds based on protonated amines and imines (Berrah *et al.* 2011, 2005*a*,*b*; Bouacida *et al.* 2005,2009), we present here the structure of Bis (2-Amino-3-carboxypyrazin-1-ium) sulfate dihydrate.

The asymmetric units of (I) includes two symmetry- independent cations and water molecules, and one sulfate anion. Cations and anions are interconnected to form *R*_3_^3^(10) and *R*_2_^2^(8) ring motifs (Etter *et al.*, 1990; Bernstein *et al.*, 1995) (Fig 1). Bond lengths and angles are as expected (Berrah *et al.* 2011; Dobson & Gerkin, 1996).

The three-dimensional structure of (I), results from undulating sheets of cations dimmers parallel to (011) plane(Fig.2 and Fig.3 )and sulfate-water chains extending along [100](Fig.3). An interesting hydrogen bonds network, in which all potential donors and acceptors are involved, and especially marked by the presence of *R*_6_^6^(26)and *R*_2_^3^(10) set-graph motifs (Etter *et al.*, 1990; Bernstein *et al.*, 1995), ensures the coherence of the structure(Fig.2 and Fig.3, table 1). This later is reinforced by the contribution of π-π, S—O··· π and C—O··· π interactions (Dorn *et al.* 2005; Janiak, 2000) (table 2,3).

### Experimental

The title compound was synthesized by reacting 3-amino-pyrazine 2- carboxylic acid with some excess of sulphiric acid in aqueous solution. Slow evaporation leads to well crystallized yellow needles.

### Refinement

All non-H atoms were refined with anisotropic atomic displacement parameters. H atoms of water molecule were located in difference Fourier maps and treated as riding on their parent oxygen atoms with O—H = 0.85, H···H = 1.40 and *U*_iso_(H) = 1.5*U*_eq_(O). The remaining H atoms were localized on Fourier maps but introduced in calculated positions and treated as riding on their parent atoms (C, N or O) with C—H = 0.95 Å, O—H = 0.84 Å and N—H = 0.88 Å with *U*_iso_(H) = 1.2*U*_eq_(C or N) and *U*_iso_(H = 1.5 *U*_eq_(O).

### Crystal data

**Table:**

2C_5_H_6_N_3_O_2_^+^·SO_4_^2^^−^·2H_2_O	*F*(000) = 856
*M**_r_* = 412.36	*D*_x_ = 1.706 Mg m^−^^3^
Monoclinic, *P*2_1_/*a*	Mo *K*α radiation, λ = 0.71073 Å
*a* = 7.7214 (4) Å	Cell parameters from 5179 reflections
*b* = 20.7043 (14) Å	θ = 2.8–27.5°
*c* = 10.6398 (7) Å	µ = 0.27 mm^−^^1^
β = 109.299 (2)°	*T* = 150 K
*V* = 1605.36 (17) Å^3^	Prism, yellow
*Z* = 4	0.55 × 0.36 × 0.15 mm

### Data collection

**Table:**

Bruker APEXII diffractometer	3146 reflections with *I* > 2σ(*I*)
graphite	*R*_int_ = 0.039
CCD rotation images, thin slices scans	θ_max_ = 27.6°, θ_min_ = 2.3°
Absorption correction: multi-scan (*SADABS*; Sheldrick, 2002)	*h* = −8→9
*T*_min_ = 0.708, *T*_max_ = 0.960	*k* = −26→26
13466 measured reflections	*l* = −13→13
3675 independent reflections	

### Refinement

**Table:**

Refinement on *F*^2^	Primary atom site location: structure-invariant direct methods
Least-squares matrix: full	Secondary atom site location: difference Fourier map
*R*[*F*^2^ > 2σ(*F*^2^)] = 0.035	Hydrogen site location: inferred from neighbouring sites
*wR*(*F*^2^) = 0.096	H-atom parameters constrained
*S* = 1.03	*w* = 1/[σ^2^(*F*_o_^2^) + (0.0491*P*)^2^ + 0.7394*P*] where *P* = (*F*_o_^2^ + 2*F*_c_^2^)/3
3675 reflections	(Δ/σ)_max_ = 0.001
246 parameters	Δρ_max_ = 0.47 e Å^−^^3^
0 restraints	Δρ_min_ = −0.48 e Å^−^^3^

### Special details

**Table:**

Geometry. All e.s.d.'s (except the e.s.d. in the dihedral angle between two l.s. planes) are estimated using the full covariance matrix. The cell e.s.d.'s are taken into account individually in the estimation of e.s.d.'s in distances, angles and torsion angles; correlations between e.s.d.'s in cell parameters are only used when they are defined by crystal symmetry. An approximate (isotropic) treatment of cell e.s.d.'s is used for estimating e.s.d.'s involving l.s. planes.
Refinement. Refinement of *F*^2^ against ALL reflections. The weighted *R*-factor *wR* and goodness of fit *S* are based on *F*^2^, conventional *R*-factors *R* are based on *F*, with *F* set to zero for negative *F*^2^. The threshold expression of *F*^2^ > σ(*F*^2^) is used only for calculating *R*-factors(gt) *etc*. and is not relevant to the choice of reflections for refinement. *R*-factors based on *F*^2^ are statistically about twice as large as those based on *F*, and *R*- factors based on ALL data will be even larger.

### Fractional atomic coordinates and isotropic or equivalent isotropic displacement parameters (Å^2^)

**Table:**

	*x*	*y*	*z*	*U*_iso_*/*U*_eq_	
O5A	0.17659 (16)	0.04617 (6)	0.03501 (11)	0.0221 (3)	
H5A	0.1515	0.0732	−0.0273	0.033*	
O6A	0.03846 (18)	0.11842 (6)	0.12557 (11)	0.0258 (3)	
N1A	0.0567 (2)	0.10587 (7)	0.37979 (13)	0.0231 (3)	
H1A1	0.0399	0.1167	0.4549	0.028*	
H1A2	0.0122	0.1303	0.3087	0.028*	
N2A	0.21592 (19)	0.01604 (6)	0.48490 (13)	0.0195 (3)	
H2A	0.1989	0.0288	0.5588	0.023*	
N3A	0.27826 (19)	−0.02410 (6)	0.25952 (13)	0.0194 (3)	
C1A	0.1266 (2)	0.06905 (7)	0.13275 (15)	0.0185 (3)	
C2A	0.1875 (2)	0.02980 (7)	0.25829 (15)	0.0177 (3)	
C3A	0.1485 (2)	0.05305 (7)	0.37379 (15)	0.0183 (3)	
C4A	0.3079 (2)	−0.03946 (8)	0.48622 (16)	0.0207 (3)	
H4A	0.3518	−0.0644	0.5654	0.025*	
C5A	0.3374 (2)	−0.05941 (8)	0.37264 (16)	0.0214 (3)	
H5C	0.4006	−0.0988	0.3731	0.026*	
O5B	−0.03794 (19)	0.32977 (6)	1.37046 (12)	0.0273 (3)	
H5B	−0.0768	0.3055	1.4185	0.041*	
O6B	−0.03786 (17)	0.23824 (5)	1.25812 (11)	0.0241 (3)	
N1B	0.0619 (2)	0.24559 (7)	1.03668 (14)	0.0238 (3)	
H1B1	0.0821	0.2313	0.9649	0.029*	
H1B2	0.0315	0.2184	1.0895	0.029*	
N2B	0.12393 (19)	0.34886 (7)	0.98346 (13)	0.0205 (3)	
H2B	0.1422	0.3332	0.912	0.025*	
N3B	0.07073 (19)	0.39911 (6)	1.20349 (13)	0.0206 (3)	
C1B	−0.0126 (2)	0.29617 (8)	1.27448 (15)	0.0196 (3)	
C2B	0.0482 (2)	0.33663 (7)	1.17934 (14)	0.0178 (3)	
C3B	0.0773 (2)	0.30786 (8)	1.06522 (15)	0.0187 (3)	
C4B	0.1438 (2)	0.41269 (8)	1.00633 (16)	0.0227 (3)	
H4B	0.1752	0.4403	0.9458	0.027*	
C5B	0.1180 (2)	0.43737 (8)	1.11810 (16)	0.0233 (3)	
H5D	0.1338	0.4824	1.1357	0.028*	
S1	0.15317 (5)	0.131125 (18)	0.73117 (4)	0.01813 (11)	
O1	0.2146 (2)	0.06509 (6)	0.71883 (12)	0.0337 (3)	
O2	0.09536 (18)	0.13565 (6)	0.85065 (12)	0.0262 (3)	
O3	0.30149 (17)	0.17773 (7)	0.74478 (12)	0.0306 (3)	
O4	−0.00482 (16)	0.14708 (6)	0.61180 (11)	0.0236 (3)	
O1W	0.23309 (17)	0.30737 (6)	0.78406 (12)	0.0262 (3)	
H1W	0.2465	0.2676	0.7697	0.039*	
H2W	0.3352	0.3253	0.7903	0.039*	
O2W	−0.15818 (19)	0.26453 (6)	1.52193 (12)	0.0296 (3)	
H3W	−0.1068	0.2285	1.5511	0.044*	
H4W	−0.1653	0.2863	1.5886	0.044*	

### Atomic displacement parameters (Å^2^)

**Table:**

	*U*^11^	*U*^22^	*U*^33^	*U*^12^	*U*^13^	*U*^23^
O5A	0.0332 (7)	0.0213 (6)	0.0140 (5)	0.0044 (5)	0.0109 (5)	0.0030 (4)
O6A	0.0400 (7)	0.0206 (6)	0.0184 (6)	0.0076 (5)	0.0115 (5)	0.0036 (4)
N1A	0.0340 (8)	0.0227 (7)	0.0140 (6)	0.0063 (6)	0.0100 (6)	0.0012 (5)
N2A	0.0269 (7)	0.0193 (6)	0.0128 (6)	−0.0002 (5)	0.0072 (5)	0.0001 (5)
N3A	0.0253 (7)	0.0168 (6)	0.0158 (6)	−0.0013 (5)	0.0063 (5)	−0.0003 (5)
C1A	0.0232 (8)	0.0172 (7)	0.0153 (7)	−0.0026 (6)	0.0065 (6)	−0.0007 (6)
C2A	0.0225 (8)	0.0159 (7)	0.0154 (7)	−0.0020 (6)	0.0072 (6)	−0.0008 (6)
C3A	0.0229 (8)	0.0175 (7)	0.0144 (7)	−0.0027 (6)	0.0062 (6)	−0.0007 (6)
C4A	0.0259 (8)	0.0177 (7)	0.0168 (7)	−0.0016 (6)	0.0048 (6)	0.0035 (6)
C5A	0.0279 (9)	0.0163 (7)	0.0186 (7)	0.0011 (6)	0.0056 (6)	0.0000 (6)
O5B	0.0480 (8)	0.0198 (6)	0.0201 (6)	−0.0009 (5)	0.0194 (5)	−0.0004 (5)
O6B	0.0355 (7)	0.0185 (6)	0.0193 (6)	−0.0015 (5)	0.0104 (5)	0.0003 (4)
N1B	0.0372 (8)	0.0188 (7)	0.0167 (6)	−0.0035 (6)	0.0105 (6)	−0.0037 (5)
N2B	0.0267 (7)	0.0221 (7)	0.0128 (6)	−0.0027 (5)	0.0069 (5)	−0.0024 (5)
N3B	0.0267 (7)	0.0172 (6)	0.0167 (6)	0.0005 (5)	0.0056 (5)	−0.0008 (5)
C1B	0.0231 (8)	0.0198 (8)	0.0141 (7)	0.0019 (6)	0.0039 (6)	0.0008 (6)
C2B	0.0216 (8)	0.0173 (7)	0.0127 (7)	0.0005 (6)	0.0033 (6)	0.0001 (5)
C3B	0.0203 (8)	0.0194 (7)	0.0142 (7)	−0.0010 (6)	0.0029 (6)	−0.0014 (6)
C4B	0.0276 (9)	0.0201 (8)	0.0195 (7)	−0.0035 (6)	0.0068 (6)	0.0020 (6)
C5B	0.0324 (9)	0.0177 (7)	0.0193 (7)	−0.0019 (6)	0.0077 (7)	0.0004 (6)
S1	0.0259 (2)	0.01758 (19)	0.01276 (18)	0.00290 (14)	0.00883 (15)	0.00224 (13)
O1	0.0605 (9)	0.0245 (6)	0.0186 (6)	0.0188 (6)	0.0163 (6)	0.0047 (5)
O2	0.0429 (7)	0.0230 (6)	0.0193 (6)	0.0051 (5)	0.0192 (5)	0.0035 (5)
O3	0.0291 (7)	0.0384 (7)	0.0238 (6)	−0.0067 (5)	0.0080 (5)	0.0029 (5)
O4	0.0266 (6)	0.0246 (6)	0.0179 (6)	0.0031 (5)	0.0049 (5)	0.0017 (4)
O1W	0.0294 (6)	0.0273 (6)	0.0252 (6)	−0.0009 (5)	0.0136 (5)	−0.0005 (5)
O2W	0.0460 (8)	0.0244 (6)	0.0229 (6)	0.0086 (5)	0.0174 (6)	0.0061 (5)

### Geometric parameters (Å, °)

**Table:**

O5A—C1A	1.3116 (19)	N1B—H1B1	0.88
O5A—H5A	0.84	N1B—H1B2	0.88
O6A—C1A	1.216 (2)	N2B—C4B	1.343 (2)
N1A—C3A	1.316 (2)	N2B—C3B	1.347 (2)
N1A—H1A1	0.88	N2B—H2B	0.88
N1A—H1A2	0.88	N3B—C2B	1.319 (2)
N2A—C4A	1.349 (2)	N3B—C5B	1.344 (2)
N2A—C3A	1.360 (2)	C1B—C2B	1.503 (2)
N2A—H2A	0.88	C2B—C3B	1.435 (2)
N3A—C2A	1.315 (2)	C4B—C5B	1.368 (2)
N3A—C5A	1.352 (2)	C4B—H4B	0.95
C1A—C2A	1.500 (2)	C5B—H5D	0.95
C2A—C3A	1.442 (2)	S1—O1	1.4670 (12)
C4A—C5A	1.365 (2)	S1—O3	1.4677 (13)
C4A—H4A	0.95	S1—O4	1.4786 (12)
C5A—H5C	0.95	S1—O2	1.4831 (12)
O5B—C1B	1.3028 (19)	O1W—H1W	0.8491
O5B—H5B	0.84	O1W—H2W	0.8542
O6B—C1B	1.218 (2)	O2W—H3W	0.8543
N1B—C3B	1.321 (2)	O2W—H4W	0.8582
			
C1A—O5A—H5A	109.5	C4B—N2B—C3B	122.81 (14)
C3A—N1A—H1A1	120	C4B—N2B—H2B	118.6
C3A—N1A—H1A2	120	C3B—N2B—H2B	118.6
H1A1—N1A—H1A2	120	C2B—N3B—C5B	119.63 (14)
C4A—N2A—C3A	122.57 (14)	O6B—C1B—O5B	125.38 (15)
C4A—N2A—H2A	118.7	O6B—C1B—C2B	121.55 (14)
C3A—N2A—H2A	118.7	O5B—C1B—C2B	113.05 (14)
C2A—N3A—C5A	119.32 (14)	N3B—C2B—C3B	121.62 (14)
O6A—C1A—O5A	123.99 (14)	N3B—C2B—C1B	117.76 (14)
O6A—C1A—C2A	121.03 (14)	C3B—C2B—C1B	120.62 (14)
O5A—C1A—C2A	114.98 (13)	N1B—C3B—N2B	119.31 (15)
N3A—C2A—C3A	122.39 (14)	N1B—C3B—C2B	124.91 (15)
N3A—C2A—C1A	118.54 (14)	N2B—C3B—C2B	115.78 (14)
C3A—C2A—C1A	119.06 (14)	N2B—C4B—C5B	118.98 (15)
N1A—C3A—N2A	118.94 (14)	N2B—C4B—H4B	120.5
N1A—C3A—C2A	125.91 (14)	C5B—C4B—H4B	120.5
N2A—C3A—C2A	115.15 (14)	N3B—C5B—C4B	121.17 (15)
N2A—C4A—C5A	119.33 (14)	N3B—C5B—H5D	119.4
N2A—C4A—H4A	120.3	C4B—C5B—H5D	119.4
C5A—C4A—H4A	120.3	O1—S1—O3	110.91 (9)
N3A—C5A—C4A	121.21 (15)	O1—S1—O4	109.31 (7)
N3A—C5A—H5C	119.4	O3—S1—O4	109.46 (7)
C4A—C5A—H5C	119.4	O1—S1—O2	109.47 (7)
C1B—O5B—H5B	109.5	O3—S1—O2	108.66 (7)
C3B—N1B—H1B1	120	O4—S1—O2	109.00 (7)
C3B—N1B—H1B2	120	H1W—O1W—H2W	105.7
H1B1—N1B—H1B2	120	H3W—O2W—H4W	108
			
C5A—N3A—C2A—C3A	0.1 (2)	C5B—N3B—C2B—C3B	1.6 (2)
C5A—N3A—C2A—C1A	178.70 (14)	C5B—N3B—C2B—C1B	−177.43 (14)
O6A—C1A—C2A—N3A	178.18 (15)	O6B—C1B—C2B—N3B	179.35 (15)
O5A—C1A—C2A—N3A	−2.2 (2)	O5B—C1B—C2B—N3B	0.9 (2)
O6A—C1A—C2A—C3A	−3.2 (2)	O6B—C1B—C2B—C3B	0.3 (2)
O5A—C1A—C2A—C3A	176.41 (14)	O5B—C1B—C2B—C3B	−178.12 (14)
C4A—N2A—C3A—N1A	178.56 (15)	C4B—N2B—C3B—N1B	−179.42 (15)
C4A—N2A—C3A—C2A	−1.9 (2)	C4B—N2B—C3B—C2B	0.5 (2)
N3A—C2A—C3A—N1A	−179.04 (16)	N3B—C2B—C3B—N1B	178.17 (15)
C1A—C2A—C3A—N1A	2.4 (2)	C1B—C2B—C3B—N1B	−2.9 (2)
N3A—C2A—C3A—N2A	1.5 (2)	N3B—C2B—C3B—N2B	−1.7 (2)
C1A—C2A—C3A—N2A	−177.09 (13)	C1B—C2B—C3B—N2B	177.27 (14)
C3A—N2A—C4A—C5A	0.8 (2)	C3B—N2B—C4B—C5B	0.8 (2)
C2A—N3A—C5A—C4A	−1.4 (2)	C2B—N3B—C5B—C4B	−0.2 (3)
N2A—C4A—C5A—N3A	0.9 (2)	N2B—C4B—C5B—N3B	−1.0 (3)

### Hydrogen-bond geometry (Å, °)

**Table:**

*D*—H···*A*	*D*—H	H···*A*	*D*···*A*	*D*—H···*A*
N1A—H1A1···O4	0.88	1.92	2.7970 (18)	175.
N1A—H1A2···O6A	0.88	2.04	2.6741 (18)	128.
N1A—H1A2···O6B^i^	0.88	2.30	3.0158 (18)	138.
N2A—H2A···O1	0.88	1.83	2.6915 (18)	167.
N1B—H1B1···O2	0.88	2.34	3.0827 (18)	142.
N1B—H1B2···O6B	0.88	2.08	2.7144 (19)	129.
N1B—H1B2···O6A^ii^	0.88	2.10	2.8237 (18)	139.
N2B—H2B···O1W	0.88	1.81	2.6705 (18)	167.
O5B—H5B···O2W	0.84	1.67	2.5046 (17)	174.
O5A—H5A···O2^i^	0.84	1.78	2.6192 (16)	175.
O1W—H1W···O3	0.85	1.95	2.7934 (19)	175.
O1W—H2W···O2^iii^	0.85	2.06	2.8996 (18)	167
O1W—H2W···O4^iii^	0.85	2.65	3.2856 (17)	133.
O2W—H3W···O4^ii^	0.85	1.88	2.7351 (17)	177.
O2W—H4W···O3^iv^	0.86	1.91	2.7633 (17)	171.
C4A—H4A···O5B^v^	0.95	2.58	3.320 (2)	134.
C4A—H4A···N3B^v^	0.95	2.45	3.369 (2)	163.
C4B—H4B···O5A^vi^	0.95	2.45	3.187 (2)	134.
C4B—H4B···N3A^vi^	0.95	2.44	3.347 (2)	159
C5A—H5C···O1W^vii^	0.95	2.55	3.175 (2)	124.
C5B—H5D···O1^viii^	0.95	2.35	3.192 (2)	148.

Symmetry codes: (i) *x*, *y*, *z*−1; (ii) *x*, *y*, *z*+1; (iii) *x*+1/2, −*y*+1/2, *z*; (iv) *x*−1/2, −*y*+1/2, *z*+1; (v) −*x*+1/2, *y*−1/2, −*z*+2; (vi) −*x*+1/2, *y*+1/2, −*z*+1; (vii) −*x*+1/2, *y*−1/2, −*z*+1; (viii) −*x*+1/2, *y*+1/2, −*z*+2.

### Table 2 π–π stacking interactions (Å, °)

Cg1 is the centroid of the N2A–C4A ring.

**Table:**

CgI	CgJ	CgI···CgJ^a^	α	β	γ	CgI···P(J)^b^	CgJ···P(I)^c^	Slippage
Cg1	Cg1^i^	3.9678 (9)	0	34.94	34.94	3.2528 (6)	3.2527 (6)	2.272

Symmetry codes: (i)1-x,-y,1-z Notes: a : Distance between centroids b : Perpendicular distance of CgI on ring plan J c : Perpendicular distance of CgJ on ring plan I α    = Dihedral Angle between the ring planes β    = Angle between the centroid vector CgI···CgJ and the normal to the plane I. γ    = Angle between the centroid vector CgI···CgJ and the normal to the plane J. Slippage = vertical displacement between ring centroids.

### Table 3 S—O···π and C—O···π interactions (Å, °).

Cg1 and Cg2 are the centroids of the  N2A–C4A and  N2B–C4B rings, respectively.

**Table:**

X	I	J	I···J	X–I···J	X···J
S1	O1	Cg1^i^	3.5922 (17)	91.83 (7)	3.9233 (8)
S1	O2	Cg1^i^	3.9845 (14)	76.88 (5)	3.9233 (8)
S1	O2	Cg2^ii^	3.8831 (15)	92.77 (6)	4.2231 (8)
C1A	O6A	Cg2^iii^	3.3136 (16)	125.62 (11)	4.1418 (18)

Symmetry codes: (i) -x, -y, 1-z; (ii) x, y, 1+z; (iii) x-1, y, z.
